# A comparative study of runs of homozygosity islands common among 12 Australian beef cattle breeds

**DOI:** 10.1093/jas/skag199

**Published:** 2026-07-11

**Authors:** Zeinab Manzari, Natalie K Connors, Julius H J van der Werf, David J Johnston, Mohammad H Ferdosi

**Affiliations:** AGBU, a Joint Venture of the NSW Department of Primary Industries and Regional Development, University of New England, Armidale, NSW 2351, Australia; AGBU, a Joint Venture of the NSW Department of Primary Industries and Regional Development, University of New England, Armidale, NSW 2351, Australia; School of Environmental and Rural Science, University of New England, Armidale, NSW 2351, Australia; AGBU, a Joint Venture of the NSW Department of Primary Industries and Regional Development, University of New England, Armidale, NSW 2351, Australia; AGBU, a Joint Venture of the NSW Department of Primary Industries and Regional Development, University of New England, Armidale, NSW 2351, Australia

**Keywords:** runs of homozygosity, beef cattle, selection, genomic

## Abstract

Cattle breeds exhibit phenotypic and genomic differences shaped by artificial and natural selection. Runs of homozygosity (ROH) analyses detect contiguous homozygous regions on chromosomes that arise as genomic diversity decreases over time. These regions may reflect shared ancestry within a population, selection pressures, or demographic processes. This study aimed to identify ROH profiles, common ROH islands, and their associated genes affected by them in twelve Australian beef cattle breeds (Alexandria, Angus, Brahman, Brangus, Charolais, Droughtmaster, Hereford, Kynuna, Limousin, Santa Gertrudis, Shorthorn, and Speckle Park). The dataset included 463,877 animals from the BREEDPLAN evaluation system, with marker densities ranging from 33K to 100K across breeds. An inverse relationship was observed between effective population size (Ne) and genome-wide ROH profiles, with populations exhibiting smaller Ne values generally accumulating more and longer ROH segments. The Speckle Park showed the highest amount of ROH and small Ne (121), while the Droughtmaster showed the lowest amount of ROH and high Ne (300), indicating their evolutionary backgrounds during the breeding program. Common ROH islands were identified on chromosomes 1, 3, 5, 6, 7, 8, 13, 14, 24, and 26. Common ROH islands on chromosomes 3, 8, and 13 primarily reflected founder contributions from Angus, Brahman, and Shorthorn to their admixed populations. In contrast, common ROH islands on chromosomes 1, 5, 6, 7, 14, 24, and 26 among breeds suggest convergent selection. Identified candidate genes within common ROH islands were enriched in biological processes related to adaptability, reproductive, and production traits, suggesting the effects of artificial and natural selection on economically important traits in Australian beef breeds. These results emphasize the importance of demographic history and selection pressures when interpreting ROH patterns and highlight specific genomic regions that can inform targeted breeding programs, monitor genetic diversity at the genomic segment level, and optimize genotyping array design.

## Teaser

This study identified runs of homozygosity (ROH) islands that are common across twelve Australian beef cattle breeds (admixed and pure breeds), suggesting that they originated from shared ancestors or selection processes.

## Introduction

Advances in genotyping technologies and statistical approaches over recent decades have refined our understanding of the genetic basis of adaptation and selection in livestock populations. In this regard, runs of homozygosity (ROH), as contiguous homozygous segments, have become valuable tools for studying autozygosity (inheritance of identical-by-descent loci) and inbreeding in livestock. At the population level, genomic regions where ROHs are shared by multiple individuals are known as “ROH islands” (Hervás-Rivero et al. 2024). These islands have been widely used to identify natural or artificial selection signatures in livestock (Cendron et al. 2024). They can result from a range of population genetic processes, including inbreeding, targeted or natural selection, genetic drift, and population bottlenecks that can occur in both inbred and non-inbred populations (Dixit et al. 2020). There are some genomic studies that have been performed to compare ROH islands among different cattle breeds (Falchi et al. 2023; Signer‐Hasler et al. 2023; Cendron et al. 2024; Hervás-Rivero et al. 2024; Proto et al. 2025). For example, Proto et al. (2025) analyzed ROH islands in five Italian native cattle breeds. They discovered signals on chromosomes 5, 6, and 16 that are involved in coat characteristics, environmental adaptation, and growth. In addition, Signer‐Hasler et al. (2023) reported selection signatures based on the ROH islands method on different bovine chromosomes, including chromosome 6 on different genomic regions near known genes (*KIT* and *LCORL*), explaining genetic variation in body size and coat color in 18 local breeds from six countries in Alpine regions. However, there has been limited research on Australian beef cattle breeds to detect ROH patterns and islands, for example, in Australian Angus (Mulim et al. 2025). Australian beef breeds are divided into *indicus*, *taurus*, and admixed breeds. Their genomes have been shaped through targeted and natural selection pressures, reflecting their unique evolutionary and breeding histories. This study is the first comparative and comprehensive study to detect ROH patterns and common ROH islands among 12 Australian beef cattle breeds (Alexandria, Angus, Brangus, Brahman, Charolais, Droughtmaster, Hereford, Kynuna, Limousin, Santa Gertrudis, Shorthorn, and Speckle Park) and to perform functional genomic annotation analyses to identify the gene lists and biological processes using genome-wide SNP data.

## Materials and methods

The genomic data of twelve Australian beef cattle breeds were extracted from the BREEDPLAN (Australian national beef genomic evaluation system, https://breedplan.une.edu.au/) genomic pipeline. The density of SNP chips for each breed ranged from 7K to 800K. These breeds consisted of three groups: *indicus*, *taurus*, and admixed populations. Brahman was the *Bos indicus* breed, while Angus, Charolais, Hereford, Limousin, and Shorthorn were the *Bos taurus* breeds. Alexandria (North Australian Pastoral Company—NAPCo), Brangus, Droughtmaster, Kynuna (NAPCo), Santa Gertrudis, and Speckle Park were the admixed populations. The extracted genomic data have passed several quality control criteria (Connors et al. 2017), and the genotypes for each breed were imputed separately using FImpute v3 (Sargolzaei et al. 2014) using the pedigree information. In the first run, all SNPs were combined to make a concise panel (excluding the SNP chips with density of more than 160K, as only a few individuals were genotyped with higher density), and then the missing genotypes were imputed using pedigree information. Subsequently, new genotyped individuals were imputed regularly using both the haplotype library and the pedigree, and the newly imputed phased genotypes were then added to the library for future imputation batches. Only SNPs with a missing rate of less than 90% across all individuals were considered for any downstream analysis as SNPs with a higher missing rate may not have reliable imputation accuracy. The map file was based on the Agricultural Research Service’s—University of California Davis (ARS-UCD) 1.2 reference genome assembly, and the pedigree was verified with genomic data (Ferdosi and Boerner 2014). After imputation, SNPs with minor allele frequency (MAF) less than 0.05 were removed using PLINK (Purcell et al. 2007). The sample size and SNP density for each breed after quality control of MAF are summarized in Table 1.

**Table 1:** The summary of the sample size and SNP density for different beef cattle breeds.

Breed	Code	Type breed	Sample size	SNP density (before QC)	SNP density (after QC)
**Alexandria**	xa	Admixed	11,553	112,481	99,565
**Angus**	aa	Pure	292,816	82,728	67,161
**Brahman**	bb	Pure	48,380	64,322	56,232
**Brangus**	bg	Admixed	8,226	44,627	42,881
**Charolais**	cc	Pure	5,734	62,052	61,987
**Droughtmaster**	dm	Admixed	7,674	61,762	59,103
**Hereford**	hh	Pure	59,388	82,892	77,735
**Kynuna**	xk	Admixed	4,909	52,620	51,854
**Limousin**	ll	Pure	3,810	58,377	58,342
**Santa Gertrudis**	sg	Admixed	8,244	100,542	87,332
**Shorthorn**	ss	Pure	328	33,664	33,451
**Speckle Park**	sk	Admixed	13,143	81,336	75,436

QC: quality control.

### Detection of ROH islands

ROHs were identified for each population separately using PLINK with the following consistent ROH‑calling criteria across breeds: 1) a minimum length of 1,000 kb (–homozyg-kb 1,000), 2) only one heterozygous genotype was allowed within the ROH (–homozyg-window-missing 0, –homozyg-het 0, and –homozyg-window-het 1), 3) a minimum SNP density per ROH of one SNP every 100 kb (–homozyg-density 100), 4) a maximum gap of 1,000 kb between consecutive homozygous SNPs (–homozyg-gap 1,000), and 5) the minimum number of SNPs defining an ROH per breed (–homozyg-snp) was estimated according to Lencz et al. (2007):


$$l=\log_{e}(\frac{\alpha }{\mathrm{ns}*\mathrm{ni}})/\log_{e}(1-\bar{\mathrm{het}})$$

where $n_{s}$ and $n_{i}$ are the number of SNPs in each animal and the number of animals, respectively. α set to 0.05 for the percentage of false-positive ROHs, and $\overset{-}{\mathrm{het}}$ is the mean heterozygosity across all SNPs, which is calculated from PLINK software. We considered the value of –homozyg-snp equal to –homozyg-window-snp in our study. Many studies have explored the parameters for detecting optimal ROH settings (Purfield et al. 2012; Meyermans et al. 2020; Falchi et al. 2024), but considerable challenges remain. This study reviewed several studies on ROH detection using medium- and low-density SNP arrays (Doekes et al. 2020; Gorssen et al. 2021), aiming to identify the most appropriate parameter settings.

The proportion of SNPs located within a ROH for a specific breed was determined by dividing the number of times in which a SNP appeared in a ROH by the total number of genotyped cattle of that breed. Subsequently, ROH islands were detected by selecting the top 1% SNPs that most frequently present in an ROH, based on breed-specific percentile thresholds (Gorssen et al. 2021). ROH islands were further filtered with criteria applied independently for each breed to ensure the detection of robust ROH islands: each ROH island must have a minimum of five SNPs per island and a maximum distance of less than 1 Mb between consecutive SNPs. These thresholds were implemented to account for differences in SNP density and ensure equitable comparison across breeds. In addition, SNPs that were more than 1 Mb apart were categorized as distinct ROH islands.

Demographic history and effective population size (Ne) are factors that influence ROH patterns; therefore, we estimated breed-specific Ne from linkage disequilibrium using the SNeP v1.1 program (Barbato et al. 2015) to interpret differences in ROH results among breeds. Manhattan plots and other visualization plots were created in R using the “CMplot” (https://github.com/YinLiLin/CMplot) and “ggplot2” packages (Wickham et al. 2016).

### Functional annotation

We annotated the genes within common ROH islands using the bovine genome annotation as outlined in the “biomaRt” R package (Durinck et al. 2009). Functional analysis was performed with DAVID software (https://davidbioinformatics.nih.gov/) on the detected genes to explore their biological mechanisms.

## Results

### Distribution of ROH patterns and ROH islands

**Figure 1:**
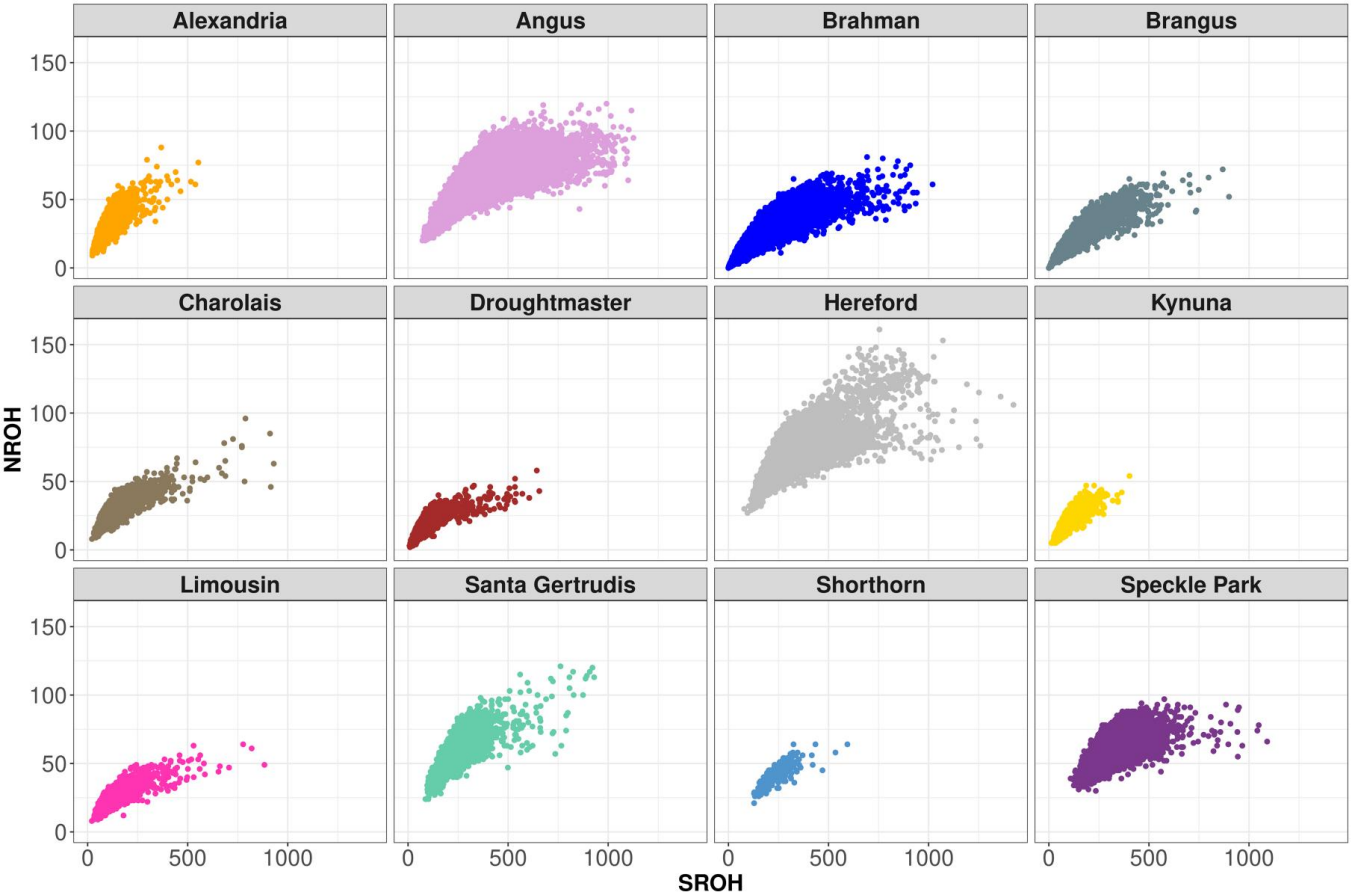
Correlation plots between N_ROH_ (number of runs of homozygosity [ROH]) and S_ROH_ (total length of ROH in mega base pairs) for 12 Australian beef cattle breeds.

**Figure 2:**
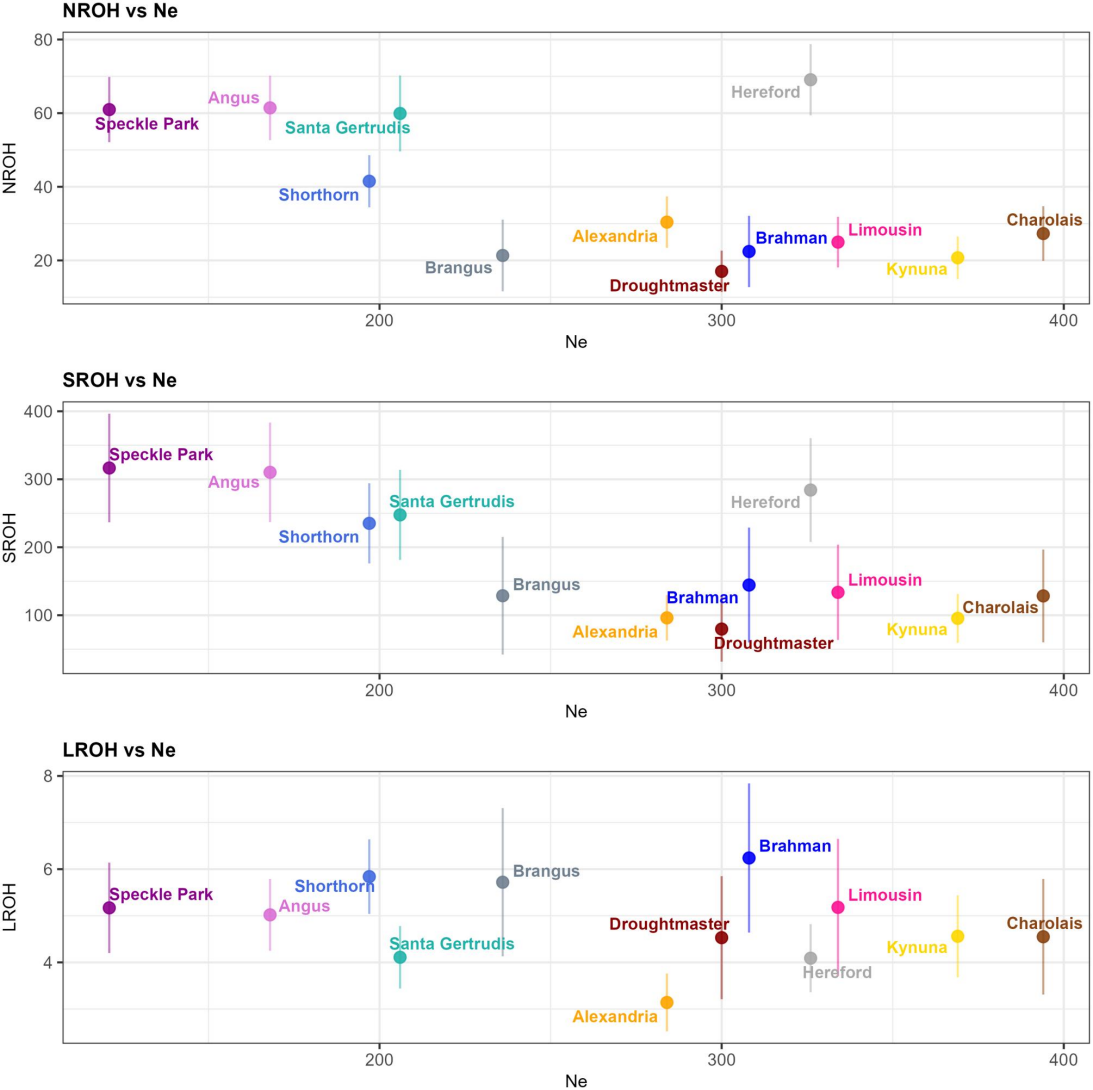
Relationship between runs of homozygosity (ROH) profiles and effective population size (Ne) across 12 Australian beef cattle breeds. Points represent mean values for each breed, with vertical error bars indicating standard deviation.

To study demographic history, we summarized ROH profiles and plotted the number of ROH (NROH) per individual against the total length of ROH (SROH) (Figure 1 and Table S1). The ROH profiles displayed different patterns among the twelve Australian beef breeds. Out of six admixed cattle breeds, Droughtmaster and Kynuna showed the smallest number and short total length in ROH. A large proportion of individuals with large numbers and lengths were identified in Angus and Speckle Park. The relationship between ROH profile and effective population size across the 12 breeds is shown in Figure 2. A consistent inverse relationship was observed between Ne and ROH profiles across the breeds. Breeds with smaller Ne values (Angus, Ne = 168; Speckle Park, Ne = 121) accumulated substantially more ROH, as reflected in higher mean NROH (61.43 ± 8.75 and 60.97 ± 8.85, respectively) and SROH values (310.21 ± 73.19 and 316.58 ± 79.81 Mb, respectively). Conversely, breeds with larger Ne values (Charolais, Ne = 394; Kynuna, Ne = 369) showed lower ROH burden, with mean NROH of 27.30 ± 7.42 and 20.72 ± 5.78, and mean SROH of 128.41 ± 68.24 and 95.44 ± 35.91 Mb, respectively.

However, notable exceptions to this inverse relationship were observed. Hereford and Santa Gertrudis exhibited high NROH (69.08 ± 9.68 and 59.91 ± 10.31, respectively) and elevated SROH values despite moderate Ne values (326 and 206, respectively). Additionally, Brahman exhibited the greatest overall average length of ROH (LROH = 6.24 ± 1.60 Mb) despite a moderate Ne of 308, substantially exceeding the LROH of breeds with lower Ne values.

**Figure 3:**
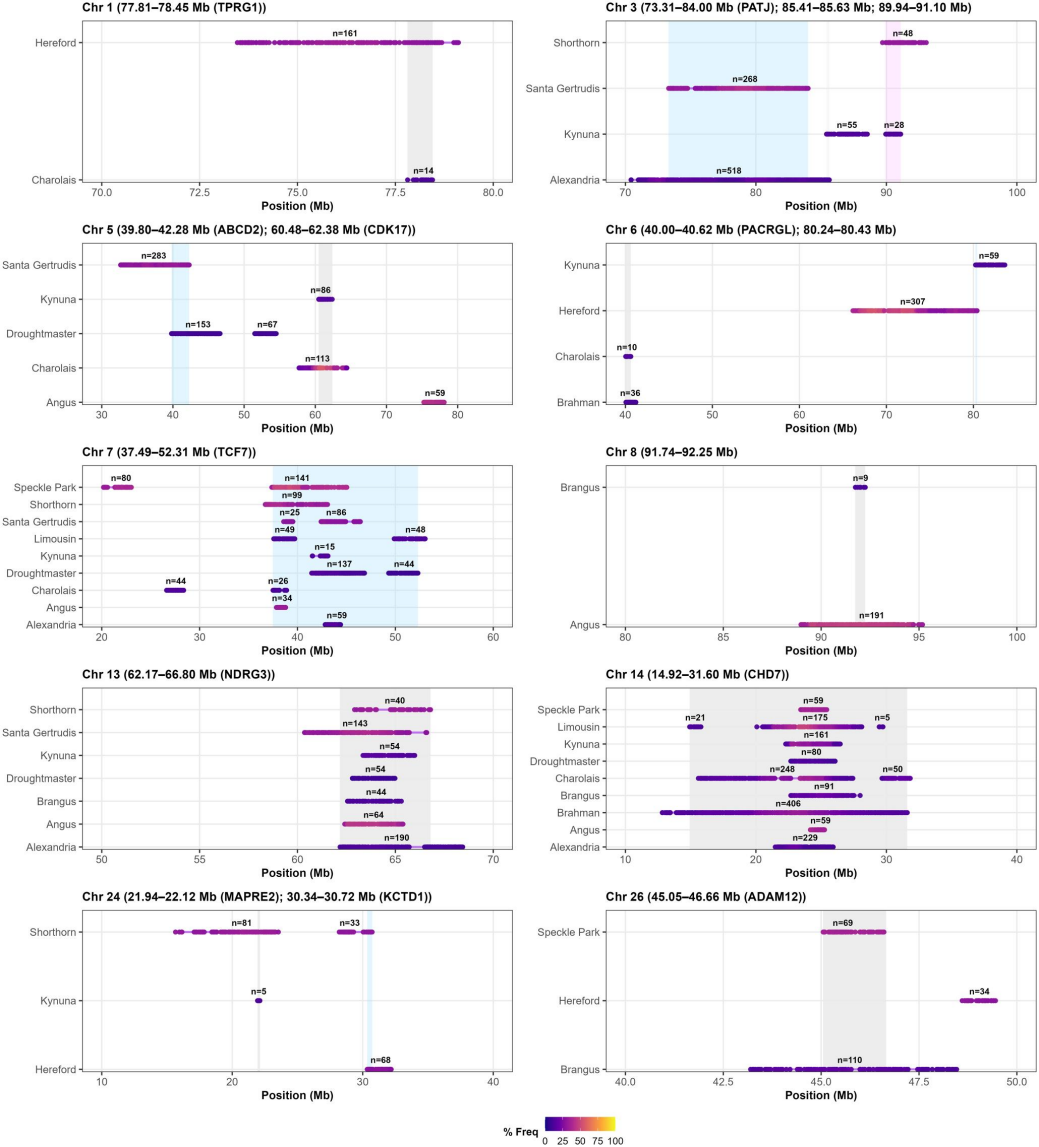
Runs of homozygosity (ROH) islands identified across 12 Australian beef cattle breeds. Horizontal segments represent ROH islands for each breed, with color intensity indicating the proportion of animals carrying each SNP within a ROH (expressed as a percentage). Some candidate genes and genomic positions of common ROH islands are listed in the header of each chromosome. The number of SNPs within each ROH island is displayed above the corresponding horizontal segment.

ROH islands were identified separately for each of the 12 Australian beef breeds (Figure S1, see online supplementary material for a color version of this figure). Then, by comparing ROH regions between breeds, several common ROH islands were identified as potential candidate regions. These common regions were located on ten different chromosomes (1, 3, 5–8, 13, 14, 24, and 26) and exceeded the 99th percentile threshold of the frequent SNPs in ROH in a population (Figure 3). In this study, “common ROH islands” refer to regions observed in at least two breeds, not necessarily in all 12 breeds. The threshold for identifying ROH islands, defined as the top 1% most frequent SNPs within ROH regions, varied among breeds due to differences in SNP density and sample size. Specifically, the percentage of SNPs of this threshold ranged from 11% (Alexandria, Brahman, Brangus, Charolais, and Limousin) to 12% (Kynuna), 26% (Hereford), 28% (Santa Gertrudis and Shorthorn), and 32% (Angus and Speckle Park) (Figure S1, see online supplementary material for a color version of this figure).

Among these populations, Kynuna cattle had the highest number (eight) of common ROH islands, whereas Brahman cattle had the lowest number (two) of common ROH islands (Figure 3). Alexandria cattle exhibited the highest number of identified SNPs (518) on chromosome 3, followed by Brahman cattle with 406 SNPs on chromosome 14, whereas Kynuna cattle with five SNPs on chromosome 24 demonstrated the lowest number of SNPs. Furthermore, the shortest length of common ROH islands was 0.18 Mb on chromosome 24 in Kynuna cattle, whereas Brahman cattle exhibited the longest common ROH islands with 18.77 Mb on chromosome 14.

**Figure 4:**
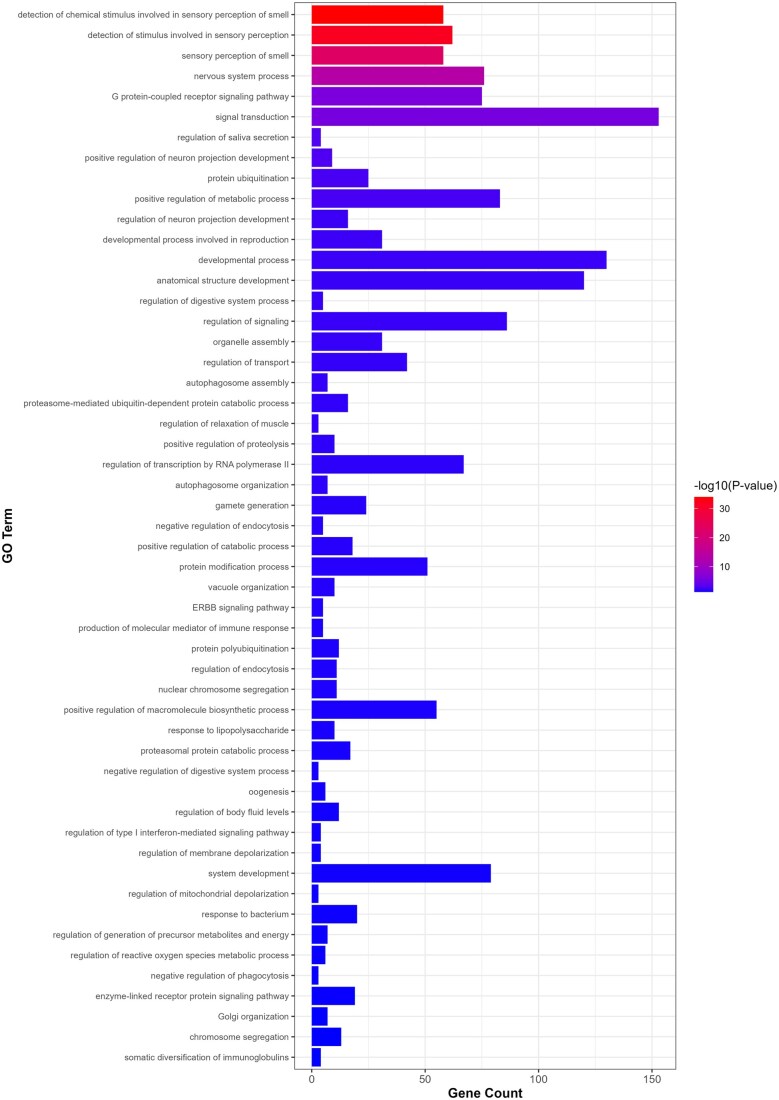
A histogram of some gene ontologies of biological processes of candidate genes on common ROH islands across beef cattle breeds.

Identifying candidate genes in common ROH islands aids in understanding their roles in economically important traits in cattle. In the common ROH islands, more than 500 candidate genes were observed among the studied breeds (Table S2). The gene ontology (GO) enrichment analysis revealed that these genes were mainly enriched in several biological processes (Figure 4). Specifically, the analyses indicated the involvement of specific genes in reproduction (eg *CHD7* and *PLAG1*) and production (eg *LEPR* and *XKR4*).

## Discussion

ROH results across 12 Australian beef cattle breeds reflected their demographic histories, effective population sizes, and breeding programs. Admixed breeds showed variable ROH patterns: some breeds, such as Alexandria, Droughtmaster, and Kynuna, exhibited low ROH consistent with admixture, while others, such as Santa Gertrudis and Speckle Park, showed elevated ROH patterns, suggesting intense selection or within-population mating. In addition, breeds with smaller Ne accumulated more and longer ROH (Angus and Speckle Park), while those with larger Ne showed fewer and shorter ROH (Charolais and Limousin). Hereford was an exception, showing high ROH despite moderate Ne, suggesting historical bottlenecks or strong selection. Common ROH islands either aligned with common ancestors (Angus, Brahman, Shorthorn) or reflected convergent selection across breeds without shared ancestry. The findings are discussed in depth in the following sections.

### An overview of ROH profile and ROH islands

The ROH patterns (number and length) provide insight into demographic history processes, including admixture, bottlenecks, and changes in effective population size (Peripolli et al. 2017). Our analysis of 12 Australian beef cattle breeds revealed distinct profiles reflecting their unique selection and breeding histories (Ma et al. 2025; Mulim et al. 2025).

Admixed populations typically exhibit fewer and shorter ROH segments compared to their founder populations due to the introduction of divergent haplotypes and the breakdown of homozygous regions through recombination (Ceballos et al. 2018). In this study, the Alexandria (mean NROH: 30.4 and mean SROH: 96.17 Mb), Droughtmaster (mean NROH: 17.03 and mean SROH: 79.63 Mb), and Kynuna (mean NROH: 20.72 and mean SROH: 95.44 Mb) exhibited characteristics of admixed populations, whereas other admixed populations revealed a high number and length of ROH due to an intensified close breeding program over time after breed formation, as seen in the Santa Gertrudis (mean NROH: 59.91 and mean SROH: 247.62 Mb) and Speckle Park (mean number: 60.97 and mean length: 316.58 Mb) (Figure 1; Table S1).

Across the dataset, an inverse relationship was consistent: breeds with smaller estimated Ne generally accumulated more frequent and longer ROH profiles. For example, Angus (Ne: 168, mean NROH: ∼60, mean SROH: ∼310 Mb) and Speckle Park (Ne: 121, mean NROH: ∼60, mean SROH: ∼320 Mb) demonstrated the highest ROH amount along with the lowest Ne (Figure 2). Conversely, populations with larger Ne tend to have fewer and shorter ROHs, such as Charolais (Ne: 394, mean NROH: 27.3, and mean SROH: 128.41 Mb) and Kynuna (Ne: 369, mean NROH: 20.72, and mean SROH: 95.44 Mb) (Figure 2; Table S1). Admixed populations and those with large Ne maintain low levels of cumulative homozygosity via gene flow, whereas populations with small Ne and those that have experienced bottlenecks may accumulate ROH through genetic drift. Mulim et al. (2025) recently investigated ROH profiles in Australian Angus; their ROH numbers were fewer than the present study, likely due to differences in sample size, SNP density, and ROH-calling parameters between the studies.

ROH length provides additional information about the timing of autozygosity: long ROH are more likely to arise from a recent common ancestry, while shorter ones indicate a more distant common ancestor (González‐Castellano et al. 2025). The L_ROH_ offers a summary statistic that illustrates the balance between the length and number of homozygous segments within the population. In our dataset, the Brahman had the largest overall average L_ROH_ value (6.24), while the lowest L_ROH_ values were found in Alexandria (3.14 Mb), Hereford (4.09 Mb), and Santa Gertrudis (4.11 Mb) (Table S1). These differences reflect distinct selection histories; the large LROH in Brahman is indicative of recent inbreeding, whereas Hereford’s greater NROH and SROH but smaller LROH suggest ancient inbreeding from historical bottlenecks or sustained selection (Manzari et al. 2026a).

In Australia, beef cattle breeding programs have been based on phenotypic selection and BLUP (Best Linear Unbiased Prediction) methodologies, and more recently, selection within these breeds has been implemented through ssGTBLUP (single-step genomic BLUP based on the T matrix) in BREEDPLAN (Johnston et al. 2023). BLUP-based selection, while effective for improving economically important traits, may increase ROH accumulation by selecting related animals as parents for the next generation. When selection intensity is high and the population size is small, the probability of selecting relatives increases, leading to elevated inbreeding and ROH accumulation. This pattern is also consistent with Hereford’s historical bottlenecks or sustained selection over extended periods. For comparison, the L_ROH_ in American Holstein (Forutan et al. 2018) and Australian Angus (Mulim et al. 2025) cattle breeds were approximately 4.54 and 5.12 Mb, respectively.

The thresholds for identifying ROH islands are influenced by several breed-specific factors, including varying levels of polymorphism, effective population sizes, and historical selection pressures (Talebi et al. 2025). The thresholds, representing the top 1% of SNPs with the highest ROH frequencies, reflect breed-specific differences in linkage disequilibrium, selection history, and genetic diversity. For example, Angus and Speckle Park breeds showed a higher threshold of ROH islands (32%) compared to others, which coincided with their smaller Ne and more ROH accumulation. Consequently, these variations in threshold values remain consistent with the demographic stability and unique genomic architecture of each breed.

### Shared ROH islands likely reflecting common ancestry

Several common ROH islands overlapped between founder breeds and their admixed populations. These ROH islands were interpreted as candidate regions that might be consistent with common ancestry, rather than evidence of inheritance of the same haplotype.

### Angus-derived breeds

The common ROH islands supported a shared genomic ancestor between the Angus and Angus-influenced admixed breeds. Common ROH islands were observed on chromosomes 7, 8, 13, and 14 in Angus and its admixed breeds, such as Brangus, Speckle Park, and Kynuna (Figure 3). For example, a common ROH island at 91.74–92.25 Mb on chromosome 8 were found in Angus and Brangus (Figure 3). Interestingly, Angus had the longest and a higher proportion of animals with SNPs in ROH islands in this region (∼47%) than Brangus (∼12%) (Figures 3; Figure S1, see online supplementary material for a color version of this figure), indicating that the ROH islands for Brangus were consistent with founder contribution (Angus) as one of its ancestors due to subsequent recombination and breed-specific demographic history. This reflects findings by Nawaz et al. (2024) and Rostamzadeh-Mahdabi et al. (2025) for Angus and Zayas et al. (2024) for Brangus, who identified this position in their ROH islands.

On chromosome 13, ROH islands were found in Angus and its admixed breeds (Brangus and Kynuna) (Figure 3). Brangus showed ROH islands in this chromosome, while no corresponding island was detected in Brahman, indicating the Angus-derived origin of this signal. These ROH islands are also reported in Angus (Cardoso et al. 2020; Kolpakov et al. 2024; Nawaz et al. 2024; Rostamzadeh-Mahdabi et al. 2025) and Brangus (Zayas et al. 2024), confirming that the candidate region is consistent with founder contribution from Angus to Brangus. Interestingly, 44% and 34% of animals with most frequent SNPs in ROH islands were reported in Angus and Shorthorn, respectively, indicating the importance of this ROH region in the two pure breeds. Kynuna is contained with an Angus (Red Angus) founder component (alongside Shorthorn, Brahman, and Tuli). The presence of this region in Kynuna further supports the persistence of Angus-derived loci across multiple admixed populations.

In contrast, on chromosome 14, a ROH island was observed in Angus, Brangus, and Speckle Park, which was also detected in Brahman, whereas no signal was found in Shorthorn (Figure 3). The reason could be highlighting an Angus-derived origin of the signal in the admixed breeds. The additional detection of a ROH island in the same interval in Brahman may reflect either preserving regions or similar selection on the same locus across breeds. Moreover, Paim et al. (2020) studied ROH islands in Brangus and founder breeds (Angus and Brahman) and found that the ROH positions found in these three breeds were similar to our results, confirming that they are consistent with both founder contributions. In addition, these homozygous regions were observed in the Angus breed (Cardoso et al. 2020; Nawaz et al. 2024; Rostamzadeh-Mahdabi et al. 2025) and in the Brangus by Zayas et al. (2024), which confirms our results.

### Brahman-derived breeds

Brahman has consistently been the primary contributor to the Australian admixed beef cattle breeds’ ability to adapt to tropical climates; therefore, common ROH islands between Brahman and Brahman-influenced admixed breeds are probably explanations for the conservation of indicine founder segments. Multi-breed overlap regions were observed on chromosome 14 in all studied breeds except for Hereford, Santa Gertrudis, and Shorthorn. It should be mentioned that Hereford showed ROH islands in another position (79.7–80.9 Mb), and Santa Gertrudis and Shorthorn did not show any ROH islands on this chromosome.

In this study, the common ROH islands on chromosome 14 at 14.92–31.60 Mb in Brahman (12.83–31.60 Mb) overlapped in multiple admixed breeds (Alexandria, Brangus, Droughtmaster, and Kynuna) (Figure 3). The presence of admixed breeds within the longer genomic interval of Brahman indicates that founder-derived homozygosity is conserved in these ROH islands. This region has been widely reported in many cattle populations and linked with multiple bovine traits (Teodoro et al. 2025; Garcia et al. 2026); thus, shared homozygosity in this region could be due to a combination of shared ancestry, selection, and demographic influences.

### Shorthorn-derived breeds

Shorthorn has clearly contributed to several admixed breeds (Alexandria, Droughtmaster, Kynuna, Santa Gertrudis, and Speckle Park). Chromosome 3 was enriched with three common ROH islands. In the first region (73.31–84.00 Mb), Alexandria and Santa Gertrudis showed more overlapping positions, reflecting their similar genetic background with Brahman and Shorthorn (Manzari et al. 2026b). The maximum proportion of animals with SNPs in ROH islands was 23% for Alexandria and 42% for Santa Gertrudis among breeds (Figure 3 and Figure S2, see online supplementary material for a color version of this figure). The second genomic region (chr 3: 85.41–85.63 Mb) was observed in Alexandria, and Kynuna populations with the smallest length. Both breeds were developed by the same breeding company (NAPCo) but with different crossbreeding programs (Manzari et al. 2026b). Although they share early ancestral stocks (Brahman and Shorthorn), subsequent breed-specific selection and introduction of different breeds have resulted in distinct genetic backgrounds, explaining the variation in ROH patterns in this region. The third genomic region (chr 3: 89.94–91.10 Mb) was found in Kynuna and Shorthorn (Figure 3). The presence of common ROH islands shows that despite genetic commonalities between the Shorthorn and Kynuna breeds (Shorthorn is known to be a founder of Kynuna), there are differences in their genomic structure. One of the most striking differences is the greater length of the ROH islands in Kynuna than in Shorthorn, which may reflect selections on this genomic region. Rostamzadeh-Mahdabi et al. (2025) also found this ROH island in Shorthorn. Thus, these results suggest that founder relationships contribute to shared homozygous regions while subsequent breed-specific demographic and selective processes shape genomic differences among populations.

On chromosome 5, a common ROH island (39.80–42.28 Mb) was identified in Santa Gertrudis and Droughtmaster (Figure 3). While neither Shorthorn nor Brahman exhibited ROH islands at this position, the presence of this shared region in these admixed breeds may reflect their common genetic background and suggests that selection or demographic processes shaped these homozygous segments following breed formation.

Chromosome 7 was enriched with common ROH islands at 37.49–52.31 Mb in taurine breeds (Angus, Charolais, Limousin, and Shorthorn) and Shorthorn-influenced admixed breeds (Alexandria, Droughtmaster, Kynuna, Santa Gertrudis, and Speckle Park), but not in Brahman, Brangus, or Hereford (Figure 3). Half of Shorthorn animals carried ROH islands in this region, consistent with previous reports (Rostamzadeh-Mahdabi et al. 2025). However, admixed breeds showed variable ROH patterns at this region, with Speckle Park displaying substantially more SNPs in the ROH island (*n* = 141) compared to its founder breeds Angus (*n* = 34) and Shorthorn (*n* = 99). These differences reflect variation in effective population size, linkage disequilibrium structure, and breed-specific demographic histories rather than simple inheritance from founder breeds. Thus, breed-specific evolutionary histories shape the frequency and position of ROH islands despite shared Shorthorn ancestry.

On chromosome 13, common ROH islands at 62.17–66.80 Mb were detected in Shorthorn and overlapped with ROH islands in Shorthorn-influenced breeds (Alexandria, Droughtmaster, Kynuna, and Santa Gertrudis), with admixed breed intervals occurring within or overlapping the Shorthorn interval (Figure 3). This pattern is consistent with preservation of a taurine founder segment followed by breed-specific drift and selection. Alexandria and Santa Gertrudis showed the greatest length of ROH islands on this chromosome. Notably, Santa Gertrudis, which has a greater genetic contribution from Shorthorn than Droughtmaster (Manzari et al. 2026b), showed more frequent SNPs in ROH islands compared to Droughtmaster. The absence of Brahman in this region supports a *Bos taurus* (Shorthorn) origin for these ROH islands, indicating that taurine genetics primarily shaped chromosome 13 ROH patterns across admixed breeds.

One common ROH island was found on chromosome 24 at 21.94–22.12 Mb in Shorthorn and Kynuna. In this ROH island, Shorthorn showed longer ROH islands than Kynuna (Figure 3). Since the Kynuna breed is an admixed breed with a shared genetic background with Shorthorn, this overlap likely reflects the genomic contribution transferred from the Shorthorn to the Kynuna during the crossbreeding process. Rostamzadeh-Mahdabi et al. (2025) reported the same ROH islands in the Shorthorn breed.

### Shared ROH islands potentially under convergent selection

Several common ROH islands were found among breeds with different breed histories, including overlaps between taurine breeds, indicine-derived breeds, and taurine-derived breeds. These overlapped ROH islands may reflect convergent selection on genomic regions influencing common production traits or environmental adaptation; however, shared ancestry and demographic processes cannot be entirely excluded. Chromosomes 1, 5, 6, 7, 14, 24, and 26 are discussed below, focusing on breed overlaps that lack common ancestry and may suggest convergent selection signals.

Charolais showed the most common ROH islands across different breeds and chromosomes, potentially indicating selection on specific genomic regions, though alternative explanations related to demographic history and linkage disequilibrium patterns should be considered, for example, on chromosome 1 (77.81–78.45 Mb) with Hereford and on chromosome 5 (60.48–62.38 Mb) with Kynuna (Figure 3). The genomic region of chromosome 5 has been previously recognized as an ROH island in Charolais (Szmatoła et al. 2019; Olšanská et al. 2020), suggesting consistent selection pressure across independent studies. Moreover, Charolais demonstrated common ROH islands with the Brahman breed on chromosome 6 at 40.00–40.62 Mb, indicating similar selective pressure for specific traits despite their distinct breed origins. This region has been previously identified in other European breeds (Szmatoła et al. 2019), suggesting it may harbor genes under widespread selection across cattle populations.

Within French cattle breeds, Charolais and Limousin showed overlapping ROH islands on chromosome 7 (37.49–38.89 Mb) and chromosome 14 (14.92–31.60 Mb), with more than half of animals in both populations carrying ROH on chromosome 14 (Figure 3). This pattern may indicate selection for similar economic traits or evolutionary convergence in response to environmental or management pressures, consistent with previous reports (Szmatoła et al. 2019; Hay et al. 2022).

Hereford showed overlapping ROH islands with other taurine breeds, particularly on chromosome 6 (80.24–80.43 Mb) shared with Kynuna, where more than half of Hereford animals carried ROH islands (∼14 Mb), potentially influenced by selection, though demographic factors and population-specific breeding practices may also contribute. Hereford also shared ROH islands with Shorthorn on chromosome 24 (30.34–30.72 Mb), consistent with selection in these British beef cattle breeds (Rostamzadeh-Mahdabi et al. 2025).

An intriguing pattern emerged on chromosome 26 (45.05–46.66 Mb), where Brangus and Speckle Park shared overlapping ROH islands, with Brangus showing longer regions than Speckle Park (Figure 3). Notably, Angus, a founder breed of both Brangus and Speckle Park, lacked ROH islands in this region, suggesting the genomic region was not inherited from Angus but may represent a relatively recent result of selection pressure after interbreeding.

### Major functional categories emerging from candidate genes

This section highlights candidate genes and GO terms within common ROH islands that have been reported in cattle genome studies. The enriched GO terms (*P*-value < 0.05) were functionally annotated to biological processes (Figure 4), indicating that candidate genes were most frequently associated with adaptability, reproductive, and growth-related traits, suggesting that common ROH islands may reflect selection pressures affecting economically important traits.

Adaptability traits: Candidate genes (*PATJ*, *TCF7*, and *TM2D1*) related to adaptability in tropical cattle breeds (Bahbahani et al. 2018; De León et al. 2019) were identified in Brahman-influenced breeds (Alexandria and Santa Gertrudis), which are raised in northern Australian subtropical and tropical environments. This pattern is consistent with indicine founder contributions or environmental selection pressures in tropical climates. Several genes were enriched for GO terms related to immune responses and environmental adaptation, including production of molecular mediator of immune response (GO : 0002440), response to bacterium (GO : 0009617), nervous system process (GO : 0050877), regulation of saliva secretion (GO : 0046877), and sensory perception of smell (GO : 0007608), suggesting selection for traits important in tropical environments.

Reproductive traits: Multiple reproductive candidate genes (*CHD7*, *MAPRE2*, *KCTD1*, *ARL10*, *PLAG1*, and *UNC5A*) were enriched in common ROH islands and have been linked to reproductive traits in cattle (Cardoso et al. 2020; Lu et al. 2021; Gangwar et al. 2024; Rahman et al. 2026). Associated GO terms included “developmental process involved in reproduction,” “gamete generation,” and “oogenesis,” suggesting strong selection pressure on reproductive pathways across Australian beef cattle populations.

Production traits: Several genes were associated with beef production traits. *LEPR* (leptin receptor) has been widely linked to body composition and feed intake/efficiency across cattle populations (Lindholm-Perry et al. 2011, 2013; Zhang et al. 2016; Smith et al. 2022; Majeres et al. 2024). *ADAM12* has been reported to play a key role in body stature (Gualdrón Duarte et al. 2023), and *XKR4* has been associated with feed efficiency and growth traits in cattle (Bejarano et al. 2023; Neto et al. 2026). Additional GO terms related to production included “positive regulation of metabolic process,” “regulation of relaxation of muscle,” and “anatomical structure development.”

Overall, these functional annotations highlight important biological processes within regions of shared homozygosity, with candidate genes enriched in pathways affecting adaptability, reproduction, and production traits in Australian beef cattle.

### Limitations of this study and the future of ROH studies of Australian beef cattle breeds

Although the present study has shown valuable results across Australian beef cattle breeds, there are some limitations in the research design that should be considered. Genotypes were sequenced using multiple SNP panels and imputed within breed, resulting in breed-specific marker sets (approximately 33K–100K SNPs across breeds; Table 1). Ideally, comparisons of ROH islands across breeds would be investigated using a common marker set. However, the SNPs shared across all breeds in this dataset were too sparse to support robust ROH calling while maintaining adequate genome coverage and the SNP-density requirements used for ROH analysis. Consequently, ROH analyses were identified within each breed using consistent ROH-calling criteria (including a minimum ROH length, SNP-density constraints, and number of SNPs in each ROH island), and comparisons of ROH results were based on overlaps in genomic regions across breeds. Ferenčaković et al. (2013) investigated the effects of SNP density and genotyping errors in autozygosity studies and found that the low SNP density array (50K) may underestimate the number of segments with short lengths. The resolution of ROH analysis also depends on SNP density (Moghbeli Damane et al. 2025). In addition, genotype imputation directly affects ROH detection by predicting missing genotypes and offering a cost-effective method to increase the number of individuals with genotypes, thereby enhancing the reliability of ROH analysis. Many factors, such as the reference panel size and SNP density, have impacts on imputation accuracy (Phocas 2022). Although most of the studied breeds in this research had very high imputation accuracy to obtain reliable genotype calls (Ferdosi and Connors 2019; Wahinya and Ferdosi 2023), sample sizes were different across breeds (Table 1). That may have affected the accuracy of imputation, particularly for Shorthorn (*n* = 328). Therefore, ROH estimates for Shorthorn should be interpreted with caution.

Future studies should aim to use high-density SNP arrays or whole genome sequences to overcome these limitations, using common SNPs with sufficient number, larger sample size, which would strengthen the findings. Integrating haplotype-based analyses with ROH islands and their functional genomic annotation will be essential for advancing genomic selection strategies and optimizing the genetic potential of livestock populations.

## Conclusion

In summary, our study revealed that the occurrence of ROH profiles differed across beef breeds due to breeding programs or demographic history. While admixed populations revealed shorter and fewer ROH, breeds subjected to reduced effective population size or recent inbreeding showed more frequent and longer ROH. As a result, the ROH profile provides valuable information regarding population structure, highlighting its importance for understanding demographic history and informing breeding strategies. We identified common ROH islands related to economically important traits (adaptability, reproductive, and production) among twelve beef cattle breeds. As a result of the crossing process, some homozygous regions in admixed populations were consistent with founder contributions and shared breed history. Thus, common ROH islands among beef breeds may reflect their evolutionary history and similar breeding programs and objectives. Furthermore, these results can help identify genomic regions of potential importance for the conservation and management of genetic diversity associated with economic traits and identify positions where increasing marker density in genotyping arrays could improve the accuracy of genomic analyses.

## Supplementary Material

skag199_Supplementary_Data

## Data Availability

The raw data are not available for public access since it is commercial and contains sensitive information.

## Abbreviations

ARS-UCD: Agricultural Research Service’s-University of California Davis
GO: gene ontology
LROH: overall average length of ROH
MAF: minor allele frequency
NAPCo: North Australian Pastoral Company
Ne: effective population size
NROH: number of ROH
QC: quality control
ROH: runs of homozygosity
SROH: total length of ROH
ssGTBLUP: single-step genomic BLUP based on the T matrix
