# Supplementary material for: A comparative study of runs of homozygosity islands common among 12 Australian beef cattle breeds
Source: J Anim Sci. 2026 Jul 11;104:skag199. doi: 10.1093/jas/skag199 (PMC13394497; doi:10.1093/jas/skag199)
Supplement: skag199_Supplementary_Data [file skag199_supplementary_data.docx]

**Table S1** Summary statistics for runs of homozygosity (ROH) profile and effective population size (Ne).

| Breed | Sample size | Total number of ROH | N_ROH_ ± SD | S_ROH_ ± SD | L_ROH_ ± SD | N_e_ |
| --- | --- | --- | --- | --- | --- | --- |
| Alexandria | 11,553 | 351,155 | 30.4 ± 6.97 | 96.17 ± 33.46 | 3.14 ± 0.62 | 284 |
| Angus | 292,816 | 17,988,222 | 61.43 ± 8.75 | 310.21 ± 73.19 | 5.02 ± 0.77 | 168 |
| Brahman | 48,380 | 1,085,255 | 22.43 ± 9.68 | 144.42 ± 84.49 | 6.24 ± 1.60 | 308 |
| Brangus | 8,226 | 175,522 | 21.34 ± 9.73 | 128.66 ± 86.46 | 5.72 ± 1.59 | 236 |
| Charolais | 5,734 | 156,515 | 27.3 ± 7.42 | 128.41 ± 68.24 | 4.55 ± 1.24 | 394 |
| Droughtmaster | 7,674 | 130,726 | 17.03 ± 5.62 | 79.63 ± 47.99 | 4.53 ± 1.32 | 300 |
| Hereford | 59,388 | 4,102,469 | 69.08 ± 9.68 | 284.16 ± 76.34 | 4.09 ± 0.73 | 326 |
| Kynuna | 4,909 | 101,699 | 20.72 ± 5.78 | 95.44 ± 35.91 | 4.56 ± 0.88 | 369 |
| Limousin | 3,810 | 95,149 | 24.97 ± 6.88 | 133.69 ± 70.07 | 5.18 ± 1.47 | 334 |
| Santa Gertrudis | 8,244 | 493,900 | 59.91 ± 10.31 | 247.62 ± 66.22 | 4.11 ± 0.67 | 206 |
| Shorthorn | 328 | 13,614 | 41.51 ± 7.08 | 235.18 ± 58.94 | 5.84 ± 0.80 | 197 |
| Speckle Park | 13,143 | 801,380 | 60.97 ± 8.85 | 316.58 ± 79.81 | 5.17 ± 0.97 | 121 |

N_ROH_: number of ROH, S_ROH_: total length of ROH in mega base pairs, L_ROH_: overall average length of ROH in mega base pairs (L_ROH_ = S_ROH_/N_ROH_), N_e_: effective population size, SD: Standard Deviation

**Table S2** Candidate genes of common runs of homozygosity islands on some autosomal chromosomes of the twelve beef cattle breeds.

| **Chr** | **Position (Mb)** | **Breed** | **Genes** |
| --- | --- | --- | --- |
| 1 | 77.81 - 78.45 | Charolais,  Hereford | *TPRG1* |
| 3 | 73.31 - 84.00 | Santa Gertrudis,  Alexandria | *AK4, ALG6, ANGPTL3, ANKRD13C, ATG4C, C3H1orf141, CACHD1, CTH, DEPDC1, DIRAS3, DNAI4, DNAJC6, DOCK7, DYNLT5, EFCAB7, FOXD3, GADD45A, GNG12, IL12RB2, IL23R, INSL5, ITGB3BP, JAK1, KANK4, LEPR, LEPROT, LRRC40, LRRC7, MIER1, NEGR1, PATJ, PDE4B, PGM1, PTGER3, RAVER2, ROR1, RPE65, SERBP1, SGIP1, SLC35D1, SNORA70, SRSF11, TM2D1, UBE2U, USP1, WLS, ZRANB2* |
|  | 85.41 - 85.63 | Alexandria,  Kynuna | *-* |
|  | 89.94 - 91.10 | Shorthorn,  Kynuna | *-* |
| 5 | 39.80 - 42.28 | Droughtmaster,  Santa Gertrudis | *ABCD2, CNTN1, CPNE8, KIF21A, LRRK2, SLC2A13* |
|  | 60.48 - 62.38 | Charolais,  Kynuna | *ELK3, CDK17, CFAP54, NEDD1* |
| 6 | 40.00 - 40.62 | Brahman,  Charolais | *SLIT2, PACRGL, KCNIP4* |
|  | 80.24 - 80.43 | Hereford,  Kynuna | *-* |
| 7 | 37.49 - 52.31 | Alexandria,  Droughtmaster,  Kynuna,  Santa Gertrudis,  Speckle Park,  Limousin,  Charolais,  Speckle Park,  Angus | *ABCA7, ABHD17A, ADAMTSL5, ADAT3, AFF4, ANKHD1, APBB3, APC2, ARHGAP45, ARID3A, ARL10, ATP5F1D, ATP8B3, AZU1, B4GALT7, BRD8, BSG, BTBD2, BTNL9, C2CD4C, C7H19orf25, C7H5orf15, C7H5orf24, CAMLG, CATSPER3, CBARP, CD14, CDC23, CDC25C, CDC34, CDHR2, CDKL3, CDKN2AIPNL, CFD, CIMAP1D, CIRBP, CLK4, CLTB, CNN2, COL23A1, COMMD10, COX7B, CSNK1G2, CTNNA1, CXCL14, CXXC5, CYSTM1, DAZAP1, DBN1, DDX41, DDX46, DNAJC18, DND1, DOK3, ECSCR, EFNA2, EGR1, EIF4E1B, EIF4EBP3, ELANE, ETF1, F12, FAF2, FAM13B, FAM193B, FAM53C, FBXL21, FGF22, FGFR4, FSTL3, FSTL4, GAMT, GCSAML, GDF9, GFRA3, GPRIN1, GPX4, GRIN3B, GRK6, GZMM, HARS1, HARS2, HBEGF, HCN2, HIGD2A, HK3, HNRNPAB, HSPA4, HSPA9, IFI47, IK, IL9, JADE2, DM3B, KIF20A, KISS1R, KLF16, KLHL3, LEAP2, LECT2, LMAN2, LRRTM2, LYPD8, MACROH2A1, MADCAM1, MATR3, MBD3, MED16, MEX3D, MGAT1, MIDN, MIER2, MISP, MXD3, MYOT, N4BP3, DUFA2, NDUFS7, NEUROG1, NHP2, NLRP3, NME5, NOP16, NRG2, NSD1, ONECUT3, OR11L1, OR14P2, OR2AJ9, OR2AK3, OR2AV1, OR2AV10, OR2AV11, OR2AV13, OR2AV14, OR2AV2, OR2AZ1, OR2AZ3, OR2AZ3B, OR2B11, OR2BB11, OR2BB12, OR2C3, OR2C3B, OR2G27, OR2G28, OR2G3, OR2G6, OR2H10, OR2L13, OR2L2, OR2L2B, OR2L2C, OR2L3, OR2L3C, OR2M10, OR2M16, OR2M4, OR2O2, OR2T1, OR2T11, OR2T16, OR2T2, OR2T22, OR2T26, OR2T27, OR2T29, OR2T3, OR2T3B, OR2T3C, OR2T4, OR2T54, OR2T55, OR2T6, OR2T60, OR2T61, OR2T62, OR2T63, OR2V1, OR2V2, OR2V2D, OR2W3, OR2W3D, OR2W53, OR2Y1, OR2Z2, OR5AE3, OR5AE4, OR6AA1, OR6F1, OR9E2, PAIP2, PALM, PCDHAC2, PCDHB1, PCDHB4, PCDHB5, PCDHB6, PCDHB7, PCSK4, PDLIM7, PFDN1, PFN3, PGBD2, PHYKPL, PITX1, PKD2L2, PLPP2, PLPPR3, POLR2E, POLRMT, PPP2CA, PRELID1, PROB1, PROP1, PRR7, PRSS57, PRTN3, PSD2, PTBP1, PURA, PWWP3A, R3HDM4, RAB24, RACK1, REEP2, REEP6, REXO1, RGS14, RMND5B, RNF126, RNF44, RPS15, SAR1B, SBNO2, SCAMP4, SEC24A, SH3BP5L, SHC2, SHROOM1, SIL1, SKP1, SLBP2, SLC23A1, SLC25A48, SLC34A1, SLC35A4, SLC4A9, SMAD5, SMIM32, SMIM33, SNCB, SNORA74, SNORD63, SNORD96, SOWAHA, SPATA24, SPMAP2, SPOCK1, SRA1, STING1, STK11, TCF3, TCF7, TGFBI, TIFAB, TMCO6, TMED9, TMEM259, TPGS1, TRIM41, TRIM52, TRIM58, TRIM7, TRPC7, TSPAN17, TXNDC15, UBE2B, UBE2D2, UIMC1, UNC5A, UQCR11, UQCRQ, VDAC1, WDR18, WDR55, WNT8A, ZCCHC10, ZFP62, ZMAT2, ZNF346, ZNF354A, ZNF496, ZNF672, ZNF692* |
| 8 | 91.74 - 92.25 | Angus,  Brangus | *-* |
| 13 | 62.17 - 66.80 | Alexandria,  Angus,  Brangus,  Droughtmaster,  Kynuna,  Santa Gertrudis,  Shorthorn,  Speckle Park | *AAR2, ACSS2, ACTL10, AHCY, ASIP, BLCAP, BPIFA1, BPIFA2A, BPIFA2B, BPIFA2C, BPIFA3, BPIFB1, BPIFB2, BPIFB3, BPIFB4, BPIFB5, BPIFB6, C13H20orf144, C13H20orf173, CBFA2T2, CDK5RAP1, CEP250, CHMP4B, CNBD2, CPNE1, CTNNBL1, DLGAP4, DNMT3B, DNPH1, DSN1, DYNLRB1, E2F1, EDEM2, EIF2S2, EIF6, EPB41L1, ERGIC3, FAM83C, FER1L4, GDF5, GGT7, GHRH, GSS, ITCH, MANBAL, MAP1LC3A, MAPRE1, MMP24, MMP24OS, MROH8, MTCL2, MYH7B, MYL9, NCOA6, NDRG3, NECAB3, NFS1, NNAT, PHF20, PIGU, PROCR, PXMP4, RAB5IF, RALY, RBL1, RBM12, RBM39, ROMO1, RPN2, SAMHD1, SCAND1, SLA2, SNORA73, SNTA1, SPAG4, SRC, SUN5, TGIF2, TLDC2, TP53INP2, TRPC4AP, UQCC1, VSTM2L, ZNF341* |
| 14 | 14.92 - 31.60 | Alexandria,  Angus,  Brahman,  Brangus,  Droughtmaster,  Charolais,  Limousin,  Kynuna,  Speckle Park | *ADHFE1, ANXA13, ARFGEF1, ARMC1, ASPH, ATAD2, ATP6V1H, BHLHE22, BPNT2, C14H8orf76, CA8, CEBPD, CHCHD7, CHD7, CLVS1, CLXN, COPS5, CPA6, CRH, CSPP1, CYP7A1, CYP7B1, DERL1, DNAJC5B, FAM110B, FAM83A, FAM91A1, FBXO32, FER1L6, GGH, H3-5, HAS2, KLHL38, LYN, LYPLA1, MCM4, MCMDC2, MOS, MRPL15, MTFR1, MTSS1, MYBL1, NDUFB9, NKAIN3, NPBWR1, NSMAF, NSMCE2, NTAQ1, OPRK1, PCMTD1, PDE7A, PENK, PLAG1, PPDPFL, PPP1R42, PRKDC, RAB2A, RB1CC1, RGS20, RNF139, RP1, RPL39, RPS20, RRS1, SDCBP, SDR16C5, SDR16C6, SGK3, SNAI2, SNORA70, SNORA72, SNTG1, SOX17, SPIDR, SQLE, ST18, TATDN1, TBC1D31, TCEA1, TCF24, TGS1, TMEM65, TMEM68, TOX, TRIM55, TRMT12, TTPA, UBE2V2, UBXN2B, VCPIP1, VXN, WASHC5, XKR4, YTHDF3, ZHX1, ZHX2, ZNF572* |
| 24 | 21.94 - 22.12 | Shorthorn,  Kynuna | *MAPRE2* |
|  | 30.34 - 30.72 | Hereford,  Shorthorn | *KCTD1, TAF4B, PSMA8, SS18* |
| 26 | 45.05 - 46.66 | Brangus,  Speckle Park | *TEX36, EDRF1, UROS, BCCIP, DHX32, FANK1, ADAM12, C26H10orf90, DOCK1, INSYN2A* |


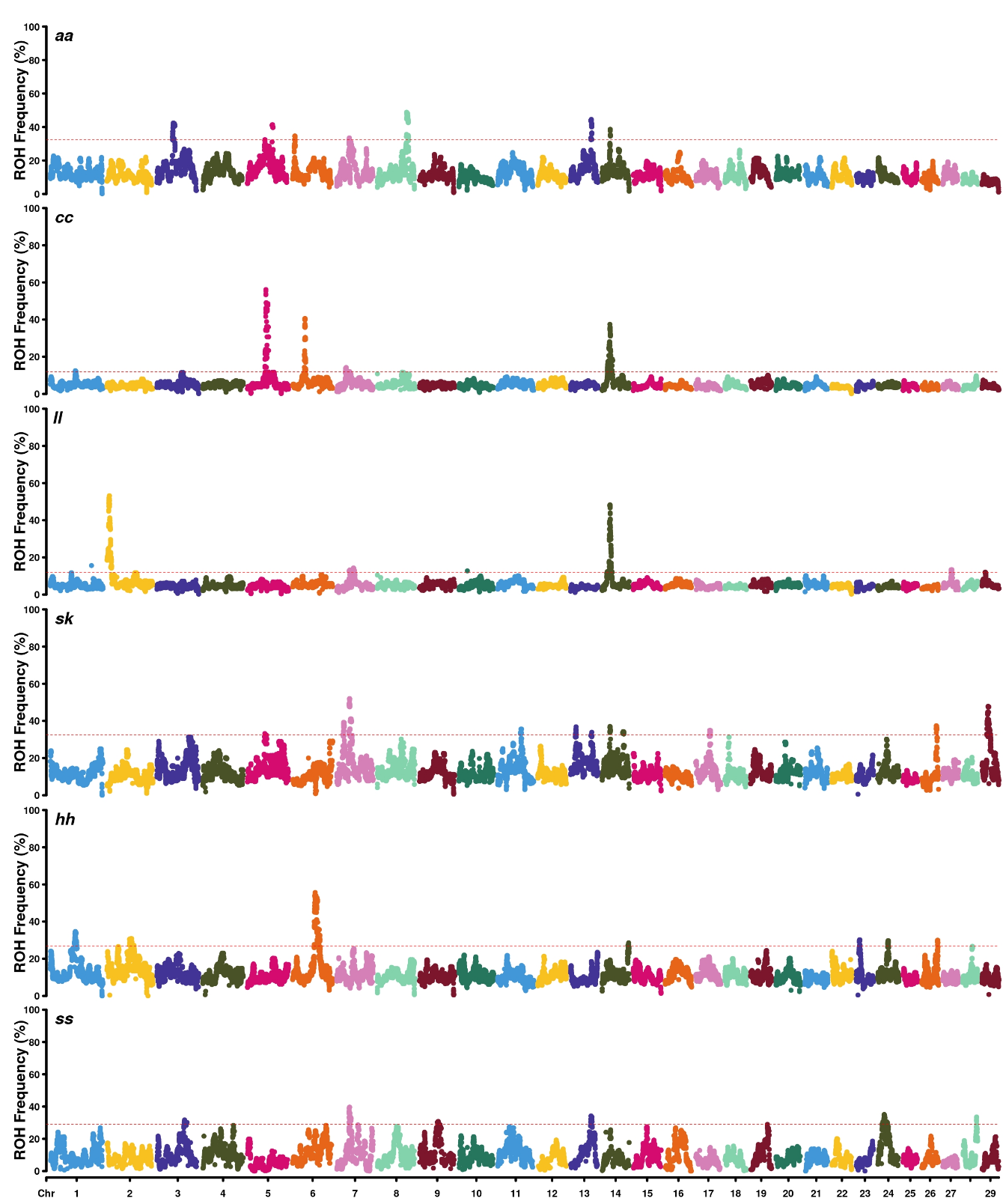


**Group A**


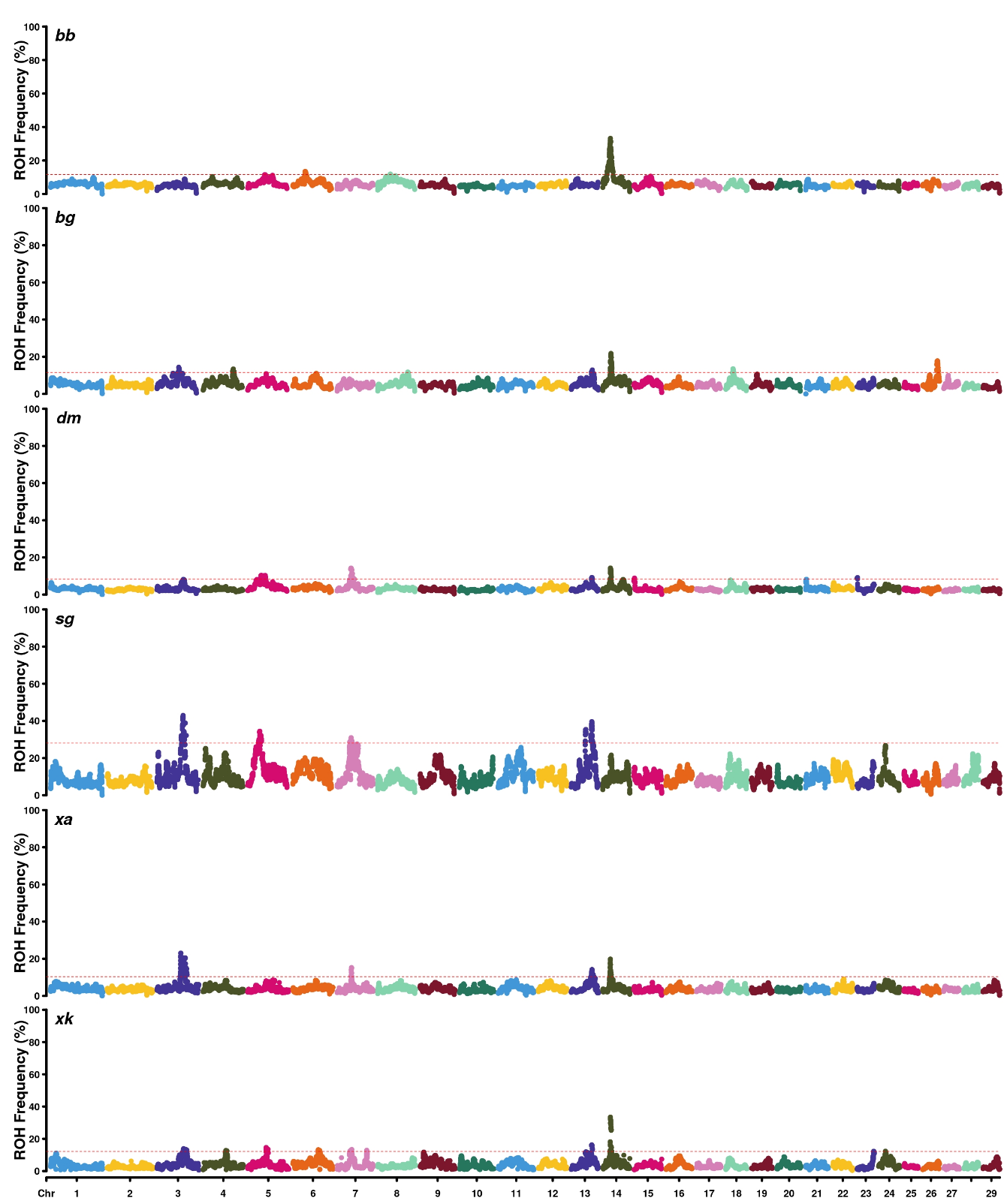


**Group B**

**Figure S1** Manhattan plot of ROH islands in twelve Australian beef cattle breeds. Group A) *Bos taurus* breeds and their admixed population: Angus (aa), Charolais (cc), Hereford (hh), Limousin (ll), Shorthorn (ss), and Speckle Park (sk). Group B) *Bos indicus* breed and its admixed populations: Alexandria (xa), Brahman (bb), Brangus (bg), Droughtmaster (dm), Kynuna (xk), Santa Gertrudis (sg). The y-axis represents the proportion of animals carrying each SNP within a ROH, expressed as a percentage. The red dashed line indicates that the top 1% of the most frequent SNPs are classified as ROH islands.
